# The landscape of long noncoding RNA during cutaneous squamous cell carcinoma progression

**DOI:** 10.1093/bjd/ljaf108

**Published:** 2025-03-27

**Authors:** Max Bone, Daniel Schreyer, Mairi Treanor-Taylor, Charlotte M Proby, Catherine A Harwood, Irene M Leigh, Peter Bailey, Gareth J Inman

**Affiliations:** School of Cancer Sciences, University of Glasgow, Glasgow, UK; Cancer Research UK Scotland Institute, Glasgow, Glasgow, UK; School of Cancer Sciences, University of Glasgow, Glasgow, UK; Cancer Research UK Scotland Institute, Glasgow, Glasgow, UK; School of Cancer Sciences, University of Glasgow, Glasgow, UK; Cancer Research UK Scotland Institute, Glasgow, Glasgow, UK; Molecular and Clinical Medicine, School of Medicine, University of Dundee, Dundee, UK; Centre for Cell Biology and Cutaneous Research, Blizzard Institute, Barts and The London School of Medicine and Dentistry, Queen Mary University of London, London, UK; Department of Dermatology, The Royal London Hospital, Barts Health NHS Trust, London, UK; Centre for Cell Biology and Cutaneous Research, Blizzard Institute, Barts and The London School of Medicine and Dentistry, Queen Mary University of London, London, UK; School of Cancer Sciences, University of Glasgow, Glasgow, UK; Botton-Champalimaud Pancreatic Cancer Centre, Lisbon, Portugal; School of Cancer Sciences, University of Glasgow, Glasgow, UK; Cancer Research UK Scotland Institute, Glasgow, Glasgow, UK

## Abstract

**Background:**

Cutaneous squamous cell carcinoma (cSCC) is a common cancer with a high morbidity rate and poor prognosis for metastatic disease. Disease may progress from premalignant actinic keratosis to invasive and metastatic cSCC, but it is perhaps best characterized as a disease continuum progressing from a differentiated to a progenitor-like state. The critical molecular mediators of this process remain poorly defined. Long noncoding (lnc)RNAs, a relatively unexplored class of RNA molecules > 200 nucleotides long, are likely to have important functional roles in cSCC.

**Objectives:**

To provide a comprehensive landscape of lncRNA expression during the cSCC continuum and to identify potentially functional lncRNA drivers of disease progression.

**Methods:**

We interrogated bulk RNA sequencing (RNAseq) data from 110 patient samples, encompassing healthy sun-exposed skin (*n* = 26), actinic keratosis (*n* = 14), primary cSCC (*n* = 66) and metastases (*n* = 4), to identify changes in lncRNA expression during disease progression. We developed a bioinformatics pipeline to infer lncRNA function based on co-expression patterns and generated a lncRNA signature score, which we validated in head-and-neck squamous cell carcinoma (HNSC) and pancreatic adenocarcinoma (PAAD). We performed bulk RNAseq on 15 patient-derived cell lines and integrated these data to identify tumour cell-specific lncRNAs and validated our findings in multiple other cSCC gene expression cohorts. Using *in vitro* knockdown approaches we investigated the functional role of *LINC00941*.

**Results:**

We found that lncRNA expression alone is sufficient to identify disease states and progression along the cSCC disease continuum. Correlation analysis revealed potentially functionally relevant lncRNAs and the processes they may regulate. We developed a 267 lncRNA signature that correlates with a progenitor-like state and predicts poor prognosis in HNSC and PAAD. Bulk RNAseq of patient-derived cell lines revealed tumour cell-specific lncRNAs, and knockdown of *LINC00941* indicated that it is required for cell proliferation and colony formation *in vitro*.

**Conclusions:**

Our findings provide a comprehensive description of lncRNA transcriptomic changes in cSCC and demonstrate their functional relevance as biomarkers and drivers of disease progression in this and, potentially, other cancers.

## Graphical Abstract

**Graphical Abstract:**
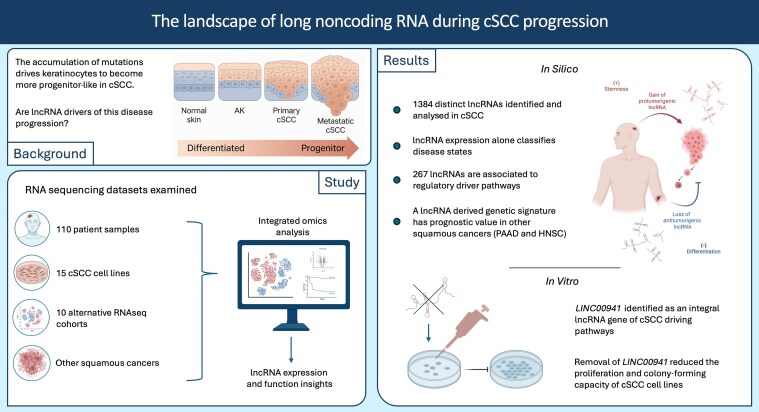

## Teaser

We provide a comprehensive landscape of long noncoding (lnc)RNA expression in cutaneous squamous cell carcinoma (cSCC) development. We identify coregulated genes, pathways and processes, and generate a 267 lncRNA signature that correlates with disease outcome in other cancers. We go on to functionally demonstrate that *LINC00941* is required for cancer cell proliferation.

What is already known about this topic?

- Long noncoding RNAs (lncRNAs) are important regulators of gene expression in normal and pathophysiological settings, including cancer.
- Cutaneous squamous cell carcinoma (cSCC) is highly prevalent, with a poor prognosis in advanced disease.
- Some lncRNAs have been identified as regulators of skin homeostasis and cancer-related molecular pathways.
- Given the estimated 100 000 lncRNA genes, our understanding of the lncRNA landscape in cSCC progression remains incomplete.

What does this study add?

- This study provides a comprehensive landscape of lncRNA expression during disease states and progression along the cSCC disease continuum in multiple cohorts.
- Patient-derived cell lines were subject to RNA sequencing, revealing novel insights into cSCC progression and lncRNA expression patterns.
- Correlation of lncRNA expression and mRNA expression reveals potential biologic regulatory modules.
- We developed a 267 lncRNA signature that correlates with cSCC progenitor status and predicts poor prognosis in head-and-neck SCC and pancreatic ductal adenocarcinoma.
- We functionally validated *LINC00941* to be required for proliferation and colony-forming ability of cSCC patient-derived cell lines.

What is the translational message?

- lncRNAs are likely to be functional regulators of cSCC disease progression.
- lncRNAs could be developed as biomarkers of disease progression.
- lncRNAs may be therapeutic targets for cSCC management.
- *LINC00941* is a promising target in cSCC and may have important roles in other cancers.

Cutaneous squamous cell carcinoma (cSCC) represents > 20% of all nonmelanoma skin cancers and is one of the most prevalent cancers globally.^1,2^ Most common in people with lighter skin phototypes and older populations, the primary risk factor for developing cSCC is exposure to ultraviolet radiation.^3^ Rates have increased annually in the UK, Australia and the USA over the last 30 years and it represents a major health economic burden.^4–6^ Although the majority of cSCCs are effectively treated with surgical resection and/or radiotherapy/chemotherapy regimens, between 1% and 5% of cSCCs metastasize, in most instances to lymph nodes, and patients with metastatic disease have a far poorer outcome, with 5-year mortality rates of > 30%.^7,8^ Studies have begun to elucidate the molecular and genetic landscape of healthy sun-exposed skin, premalignant actinic keratosis (AK), *in situ* cSCC, invasive cSCC and metastatic cSCC.^3,9–12^ Driver gene mutations can be detected in all stages of disease, and transcriptomic studies have inferred that cSCC disease progression is best described as a continuum from a differentiated to a progenitor-like state with a more progenitor-like and poorly differentiated status resulting in a greater incidence of metastasis, more invasive tumour types and worse patient outcome.^3,13–17^ The molecular basis and potentially actionable driver events of cSCC continuum progression remain incompletely defined and there is a paucity of targeted therapies for both early and advanced disease.^18^

Long noncoding RNAs (lncRNAs) are a family of RNA molecules that are > 200 nucleotides in length, have functional roles beyond encoding for proteins based on the sequence and/or structure of the molecules themselves, and are of emerging importance in cancer.^19^ lncRNAs are expressed in all cells and are predicted to make up > 68% of the human transcriptome with unique tissue-specific expression and function. They can be subcategorized based on their proximity to coding genes, including antisense, intergenic, intronic, sense overlapping and processed transcripts, and further characterized by subcellular localization, which can change between isoforms of the same lncRNA.^20–22^ lncRNAs have been found to have nuclear and cytoplasmic functions and regulate a diverse array of cellular processes during normal development, tissue homeostasis and repair, and disease pathogenesis.^23–27^ The roles and mechanisms of action for the vast majority of lncRNAs have yet to be fully elucidated.^28,29^ Recent bulk RNA sequencing (RNAseq) studies have identified several lncRNAs implicated in cSCC. *LINP1* was found to inhibit eIF2α phosphorylation via direct interaction leading to an oncogenic modulation of the unfolded protein response.^30^ The upregulation of lncRNAs such as *LINC00460* and *PICSAR* and downregulation of *NEAT1* have been found to act as drivers of proliferation in cSCC via epigenetic regulation of oncogenic pathways.^31–33^ However, as the total number of characterized lncRNAs in cSCC represent a handful of the estimated 100 000 genes and 300 000 transcripts, many novel insights remain to be elucidated by exploring these molecules.^34^

Here we sought to catalogue and identify functionally relevant lncRNAs in cSCC disease progression. We interrogated our RNAseq dataset encompassing 110 patient-derived skin samples,^3^ and identified changes in lncRNA expression between disease states and progression along the cSCC disease continuum toward a progenitor-like state. We identified 267 lncRNAs in cSCC that were differentially expressed and associated with 9 unique co-expressed networks of genes with distinct functional roles in disease progression. We developed a signature score of these 267 lncRNAs and found that this had prognostic relevance in head-and-neck squamous cell carcinoma (HNSC) and pancreatic adenocarcinoma (PAAD) datasets. Further insight into the functional roles of these lncRNAs was gained by sequencing 15 patient-derived cSCC cell lines and by *in vitro* loss-of-function analyses.

## Materials and methods

Full informatic and *in vitro* workflows are available in Appendix S1 (see Supporting Information).

### RNA sequencing

The collection of patient punch-biopsied skin samples and subsequent generation of RNAseq data were conducted as described in Bailey *et al*.^3^ Fifteen patient-derived cSCC cell lines (PDCLs) that had been previously authenticated via DNA profiling (described in Hassan *et al*.),^35^ were cultured as described below (‘Tissue culture’) and subjected to RNA isolation and sequencing as part of the same cohort (GSE266912; https://www.ncbi.nlm.nih.gov/geo/query/acc.cgi?acc=GSE266912).

### Pan-cancer RNA sequencing analysis

The batch correction and sample selection of multiple cSCC cohorts were undertaken previously by Bencomo and Lee,^36^ with full details of each study listed in their repository. The RNAseq data from 10 cohorts were obtained, with all the samples of Bailey *et al*. excluded,^3^ and subject to subsequent signature analysis in RStudio (https://github.com/rstudio/rstudio).^37–47^ Gene expression and survival data obtained from The Cancer Genome Atlas (TCGA; https://www.cancer.gov/tcga) were realigned to incorporate the most recent lncRNA transcripts obtained by the recount3 project.^48^

### Tissue culture

Human IC1 Met (cat. #153676; https://cancertools.org/) and Met-1 (Research Resource Identifier: CVCL LN09) SCC cell lines were cultured as outlined in Hassan *et al*.^35^

### Antisense oligonucleotides

Customized antisense oligonucleotides (gapmers) targeting *LINC00941* [gapmer ID 1 (LG00796994), gapmer ID 2 (LG00796996), gapmer ID 3 (LG00796997)]^49^ were designed by QIAGEN (Hilden, Germany) and transfected as per the manufacturer’s protocol.

### Quantitative reverse transcription polymerase chain reaction

Quantitative reverse transcription polymerase chain reaction (qRT-PCR) was performed using SYBR green (Thermo Fisher) on a QuantStudio^TM^ 3 machine (Thermo Fisher Scientific, Waltham, MA, USA) using the ^ΔΔ^Ct method as per the manufacturer’s protocol. Primers were designed on Primer3Plus (https://www.primer3plus.com; Appendix S1).

### Cell growth assays

Proliferation assays were performed using an IncuCyte^®^ ZOOM Live-Cell Analysis System (Sartorius, Göttingen, Germany). Colony formation assays were stained using crystal violet and counted under a Zeiss Axiostar light microscope (Carl Zeiss, Oberkochen, Germany). Statistical and graphical analyses were done with Prism version 10 (GraphPad, La Jolla, CA, USA).

## Results

### Unsupervised clustering of long noncoding RNA expression profiles predicts cutaneous squamous cell carcinoma disease state

**Figure 1:**
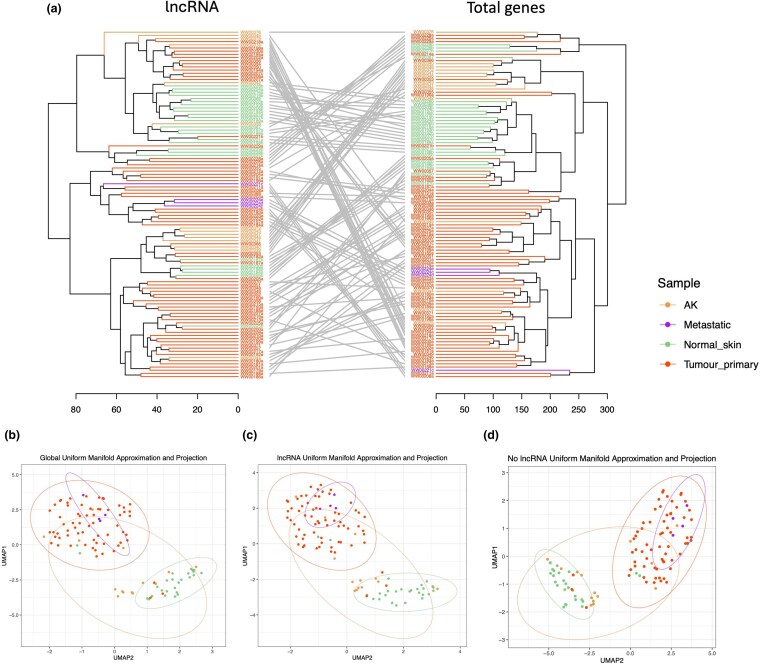
Unsupervised clustering of long noncoding (lnc)RNA and all genes involved in cutaneous squamous cell carcinoma (cSCC), and heatmap of lncRNA expression. (a) Two unsupervised hierarchical cluster dendrograms of lncRNA-only expression and all gene transcription detected in cSCC, combined as a tanglegram using the package ‘dendextend’ in R (R Foundation for Statistical Computing, Vienna, Austria). The colours of the samples match the description in the key. (b–d) Two-component uniform manifold approximation and projection (UMAP) with a random state of 15 was performed on (b) total normalized gene expression counts, (c) total lncRNA normalized counts and (d) all genes excluding lncRNA. The sample colours correspond to the description in the key. AK, actinic keratosis.

To assess the potential of lncRNAs as biomarkers and drivers of cSCC disease progression, we interrogated our bulk RNAseq data of 110 patient samples [healthy skin (*n* = 26), AK (*n* = 14), primary tumour (*n* = 66) and metastatic cSCC (*n* = 4)] for lncRNA content (Tables S1, S2; see Supporting Information).^3^ When analysing all genes, an unsupervised hierarchical clustering previously identified two main clusters, designated as class 1 and class 2, with class 2 comprising primarily samples with more advanced disease states.^3^ We identified 1384 lncRNAs in this dataset (Table S3; see Supporting Information) and performed unsupervised hierarchical clustering with all RNAs and with lncRNAs alone. Similar results were obtained with some variance, with the lncRNAs alone and the total gene expression analyses revealing two distinct groups of samples, with one containing a higher proportion of advanced disease samples (Figure 1a). Remarkably, uniform manifold approximation and projection (UMAP) of all genes and lncRNA only found that lncRNA alone complementary to total gene expression can identify the disease state of samples, with two distinct clusters arising (Figure 1b–d). One comprised primarily healthy sun-exposed skin and lower progenitor score-ranked AK and primary cSCC; the other cluster had higher progenitor score-ranked samples. Notably, metastatic samples clustered closer together in the lncRNA alone UMAP than the total gene and RNA excluding lncRNA expression UMAPs. Furthermore, disease state-specific lncRNA clustering suggested that lncRNA expression patterns change during disease state transitions (Figure S1; see Supporting Information).

### Long noncoding RNAs are differentially expressed between clinically defined disease states in cutaneous squamous cell carcinoma

**Figure 2:**
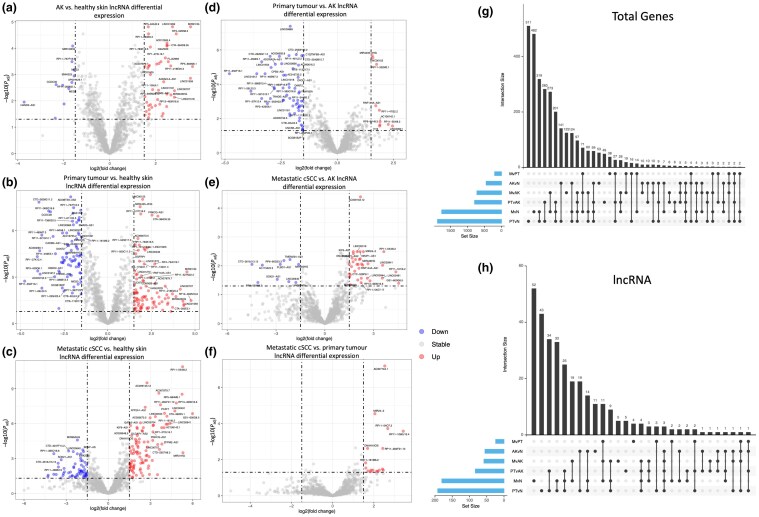
Differential gene expression and long noncoding (lnc)RNA analysis in cutaneous squamous cell carcinoma (cSCC). (a–f) Volcano plots of log2 fold change (log2FC) against log10 of the adjusted *P*-value (*P*_adj_) of the degree in change showing lncRNA alterations only. ‘Up’ (red dots) denotes an upregulation of gene expression derived from a log2FC > 1.5 and a statistical significance of *P* < 0.05. ‘Down’ (blue dots) denotes downregulated gene expression log2FC < –1.5 and a statistical significance of *P* < 0.05. ‘Stable’ (grey dots) was defined as all genes whose expression did not meet ‘up’ or ‘down’ criteria. (g) Upset plot of genes that were identified as ‘up’ or ‘down’ regulated in panels (a–f). (h) An upset plot based on (g) but containing only the lncRNA genes identified as being differentially expressed. AK, actinic keratoses; M, metastatic; N, normal; PT, primary tumour.

Differential expression analysis revealed significantly altered lncRNAs between each clinically defined sample group [Figure 2a–f; Table S4 (see Supporting Information)]. Up- and downregulated lncRNAs were identified across all comparisons of disease state. Of note, three of the five genes identified as significantly differentially expressed across all stages of cSCC disease progression in the entire transcriptome (excluding from primary tumour to metastatic disease; Figure 2g, h) were lncRNAs (*AC108142.1*, *RP11-554I8.2* and *RP13-463N16.6*). The two mRNA-encoding genes in this subset have been associated with SCC: *PTHLH* has been recognized as a promoter of tumour growth in HNSC and *AIM2* as a regulator of both growth and invasiveness of cSCC.^50,51^ These observations suggest that the three consistently differentially expressed lncRNA transcripts, *AC108142.1*, *RP11-554I8.2* and *RP13-463N16.6* – which remain functionally uncharacterized – may play direct tumorigenic roles in cSCC.

Total lncRNA expression was visualized with a heatmap (Figure S2a; see Supporting Information) and compared with our previously developed differentiated-to-progenitor (DvP) transcriptomic score,^3^ as well as the immune status of the patient samples. This revealed that the expression of lncRNAs in cSCC was not only sufficient to correlate with disease state, but also the degree to which cSCC samples are progenitor-like. Exploring lncRNAs that were differentially expressed based on a high vs. low DvP score (DvP quartiles 4 and 3 vs. DvP quartiles 2 and 1) showed a high degree of overlap with clinically defined sample comparisons [Figure S2b; Tables S4, S5 (see Supporting Information)] and indicates that lncRNAs may be fundamental to driving disease state and progenitor phenotypes in cSCC.

### Weighted correlation network analysis of differentially expressed genes and long noncoding RNAs reveals eigengene modules highly associated with advancing disease state and progenitor phenotypes

To infer potential functional roles of lncRNAs in cSCC, weighted correlation network analysis (WGCNA) was performed to identify mRNAs that were co-expressed and potentially regulated by lncRNAs. Any gene found to be differentially expressed between any cSCC clinically defined sample was used for WGCNA (Figure 2g; Table S4); a soft thresholding power of 14 and Spearman’s correlation were used as parameters for analysis (Figure S3; see Supporting Information). Groups of highly correlated genes (eigengenes) formed multiple distinct clusters that were sufficiently differentiated from one another based on thresholding parameters of these 14 modules. One was collapsed due to low count and a high overlap with a neighbouring module to form 13 final eigengene modules. These modules, which were generated based on an unsupervised correlation, were then correlated, in terms of collapsed eigengene expression, to the DvP status of all samples (Figure S4; see Supporting Information). Of the 13 eigengene modules, 9 were identified as statistically significant and correlating to the DvP score of each sample (Figure S5; see Supporting Information). Within these modules brown and blue were recognized as having the highest and the lowest correlation to DvP score, respectively, and a reduction in expression of genes in the blue module and a gain of expression of genes in the brown module as having the highest association with continually advancing disease progression.

### Expansion of eigengene modules significantly correlated with progenitor score reveals functional roles associated with cutaneous squamous cell carcinoma and networks of potential long noncoding RNA drivers of disease

**Figure 3:**
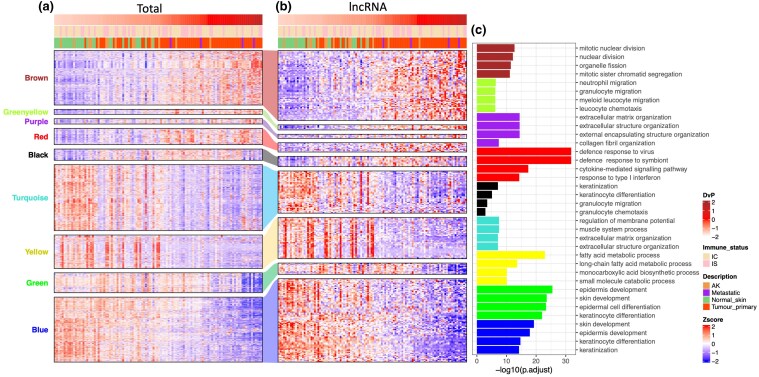
Heatmaps of eigengene module expression and filtered long noncoding (lnc)RNA expression with corresponding Gene Ontology. (a) Heatmap of gene expression of all genes within corresponding eigengene modules ordered by differentiated-to-progenitor (DvP) score. (b) Heatmap of lncRNA content only within corresponding eigengene modules, ordered by DvP score. (c) Ontology of all genes corresponding to the eigengene content in (a), the bar colours of which correspond to the names of eigengene modules. AK, actinic keratosis.

Each of the nine eigengene modules was expanded to analyse the individual expression profiles of the genes contained within them (Figure 3a). As predicted, the expanded expression profiles visually mirrored the statistically significant correlations of the collapsed modules to the progenitor score of cSCC samples [Figure 3a; Figure S5, Table S6 (see Supporting Information)]. Each of these 9 modules was then filtered for lncRNA content only (Figure 3b); all were found to contain lncRNAs (267 in total), the expression of which also shared similar trends with the total gene expression patterns. Using clusterProfiler 4.0, Gene Ontology analysis was carried out on the total eigengene content (Figure 3c). The brown eigengene module contained primarily mitosis-associated ontologies, including mitotic nuclear division, nuclear division and mitotic sister chromatid segregation. Genes in this module, including mRNA and lncRNA, are likely to drive cell proliferation and maintain the progenitor-like state. The green, yellow, red and black modules all contained immune response-associated ontologies and genes that increased in expression as cSCC became more progenitor-like. Interestingly, the black and red modules contained genes that increased and then decreased in association with DvP score (Figure S5e, g), with reduced expression of total genes and lncRNAs in the most progenitor-like samples, indicating that the increase in immune signalling through cSCC progression is reduced as the disease becomes highly advanced. The purple and turquoise modules contained genes that regulate several similar ontological processes involving cellular membranes and the extracellular environment. The gain or loss of expression of genes associated with such pathways may be essential in tumour development and increase with progenitor-like phenotypes in cSCC. The yellow module indicated that complete reduction in the expression of fatty acid metabolism-associated genes occurs as cSCC progresses and that a large proportion of genes co-expressed with these pathways are lncRNAs. The blue and green modules contained differentiation and keratinization ontologies and reflect the loss of differentiation associated with disease progression and the acquisition of a progenitor-like state.

### Functionally active long noncoding RNA signature is conserved in alternative cutaneous squamous cell carcinoma (cSCC) cohorts and cSCC cell lines

**Figure 4:**
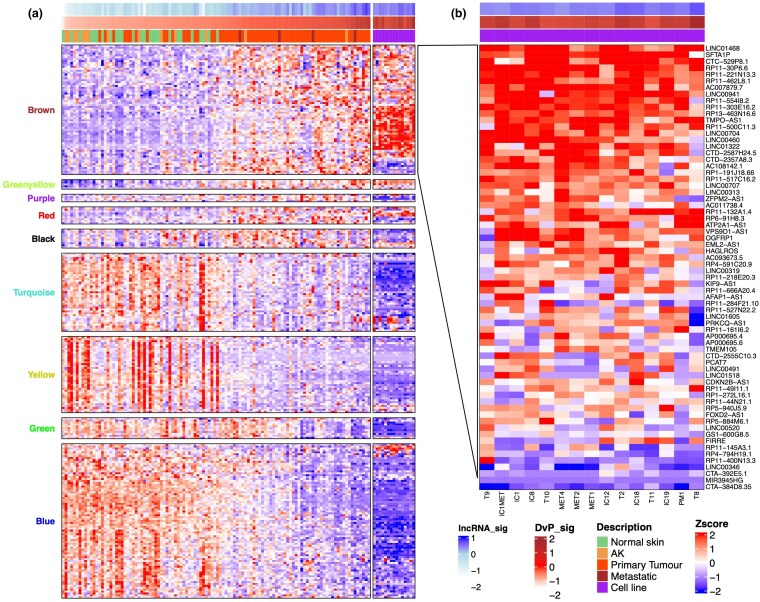
Heatmaps of eigengene module expression filtered for long noncoding (lnc)RNA expression in 15 patient-derived cutaneous squamous cell carcinoma cell lines. (a) Heatmap of lncRNA content only within corresponding eigengene modules and associated lncRNA signature score, ordered by differentiated-to-progenitor (DvP) score in patient-derived samples and separated according to cell line-derived expression data. (b) Enhanced view of the hierarchically clustered lncRNAs in the brown eigengene module across the 15 cell lines. AK, actinic keratosis.

Next, we generated a signature score for these 267 lncRNAs in the 110 patient-derived samples and 15 cSCC cell lines using ConsensusClusterPlus (Table S7; see Supporting Information). Subsequently, their expression in 15 PDCL samples was analysed alongside the previous dataset, revealing similar trends in lncRNA expression across all eigengene modules (Figure 4a). The lncRNA and DvP signature scores were high in all cell lines, further validating both as a means of predicting cSCC disease state. The majority of lncRNAs associated with proliferation showed high expression in the cell lines, providing numerous targets for future *in vitro* functional validation (Figure 4b). To further validate our generated lncRNA signature genes as drivers of cSCC progression, we explored the RNAseq data of 10 other cSCC cohorts (Figure S6; see Supporting Information),^37–47^ and revealed that both DvP and lncRNA signature scores could present a spectrum of disease progression from normal skin to higher-grade SCC samples. When exploring differentially expressed lncRNA in these bulk cohort samples, we not only found additional differentially expressed lncRNAs, but also a high degree of overlap in expression patterns of the 110 patients included in the study of Bailey *et al*. (Table S8; see Supporting Information).^3^ In the cohort of Bailey *et al*.,^3^ including the 15 PDCL and the 10 alternate cohorts of cSCC RNAseq, the DvP signature and lncRNA score of each sample were significantly correlated, with a Spearman’s rho of ≥ 0.9 in both instances (Figure S7; see Supporting Information), highlighting the association of these 267 lncRNAs with progenitor-like phenotypes and disease progression in cSCC.

### Cutaneous squamous cell carcinoma (SCC) long noncoding signature score predicts patient survival in head-and-neck SCC and pancreatic adenocarcinoma

As there is currently insufficient patient outcome data for cSCC, we chose to explore other cancers where poorly differentiated status is linked to worsened disease outcomes, to evaluate the potential prognostic use of the lncRNA signature. Using recently aligned TCGA datasets, obtained from the recount3 project repository,^48^ we investigated potential correlations with patient survival outcomes. When comparing the median and quartile-level signature scores, a statistically significant decrease in patient survival was found in both the HNSC and PAAD datasets when patients’ profiled tumours expressed high lncRNA signature scores (Figure S8; see Supporting Information). Interestingly these effects were not found in other cancers, including melanoma and squamous cell lung cancer, indicating that these lncRNAs may be tumour subtype-specific (Figure S9; see Supporting Information). The ability of the signature score to predict outcomes indicates how important the lncRNA signature score is and suggests that the lncRNAs involved may play key roles in several types of cancer.

### *LINC00941* in cutaneous squamous cell carcinoma positively regulates proliferation *in vitro*

**Figure 5:**
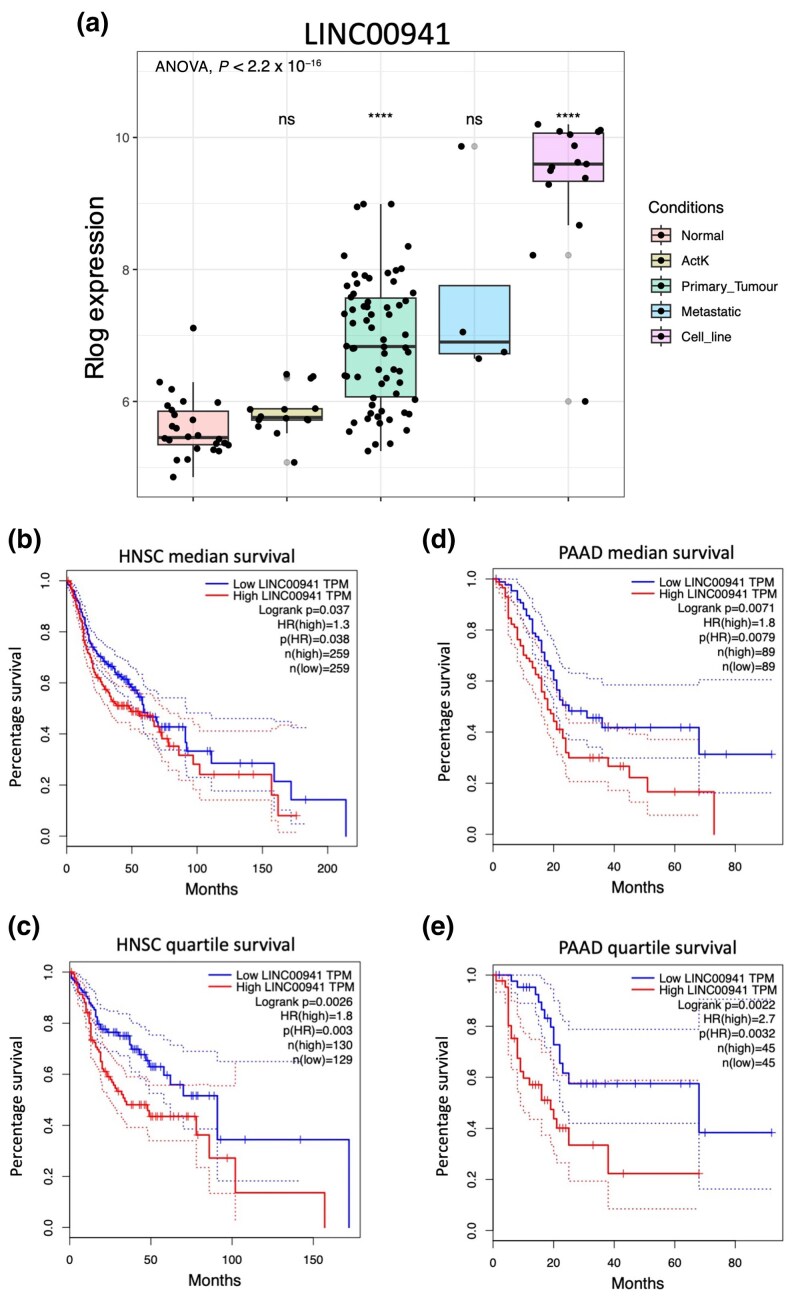
Validation of *LINC00941* as a candidate gene. (a) Relative R_log_ expression of *LINC00941* in cutaneous squamous cell carcinoma (cSCC) tissues and 15 patient-derived cSCC cell lines. Statistics calculated relative to healthy skin samples (healthy, *n* = 26; actinic keratosis, *n* = 14; primary tumour, *n* = 66; metastatic, *n* = 4; cell line, *n* = 15). Using the online platform GEPIA (http://gepia.cancer-pku.cn/), the abundance of *LINC00941* RNA was compared with the survival rates of various patient tumours. All results displayed a statistically significant inverse correlation of *LINC00941* expression to patient survival. (b) Median survival data for head-and-neck SCC (HNSC). (c) Quartile survival data for HNSC. (d) Median survival data for pancreatic adenocarcinoma (PAAD). (e) Quartile survival data for PAAD. ActK, actinic keratosis; HR, hazard ratio; ns, not statistically significant (i.e. *P* > 0.05); TPM, transcripts per million. ****P < 0.0001.

**Figure 6:**
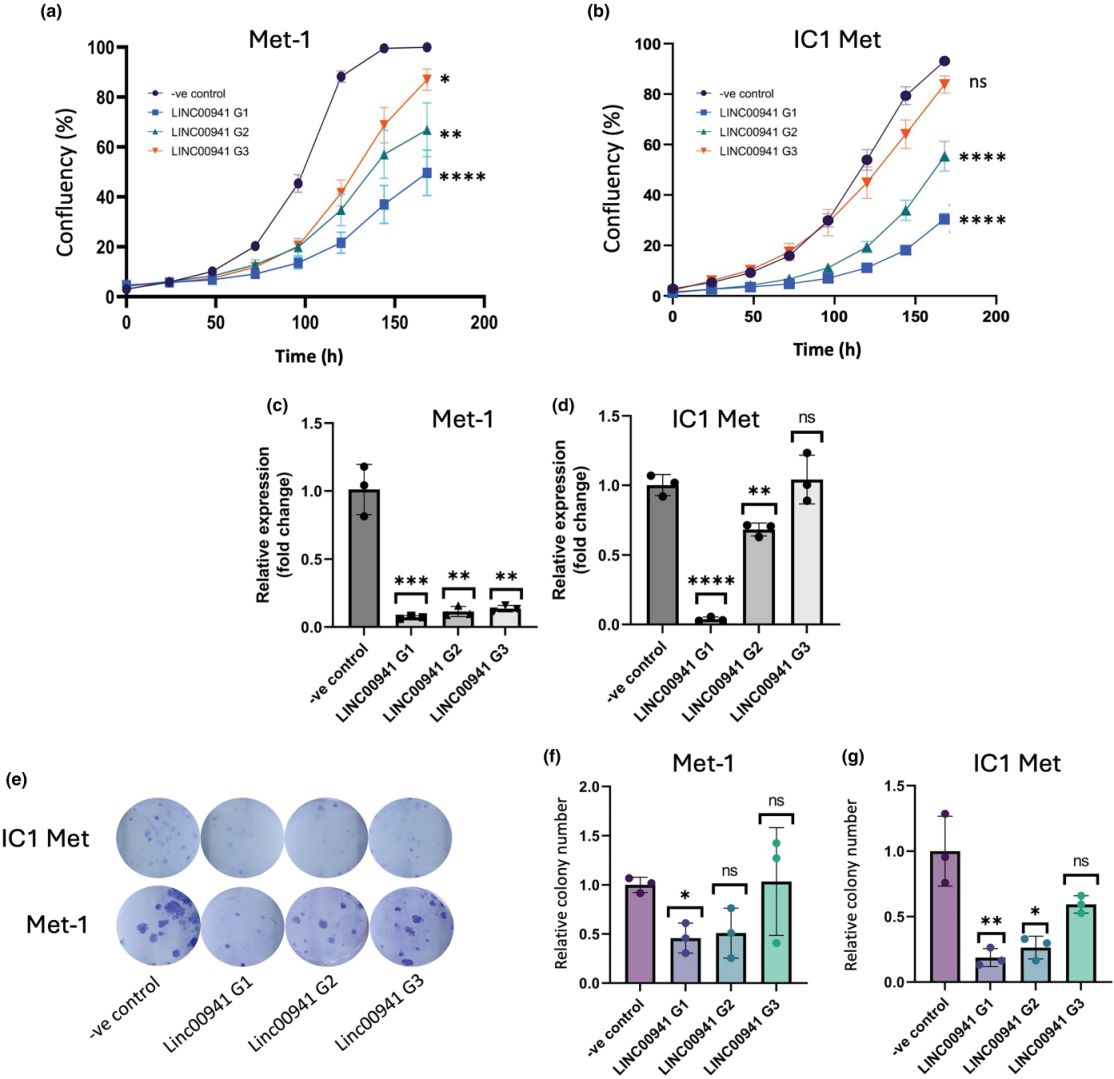
*In vitro* analysis of pro-proliferative long noncoding (lnc)RNA *LINC00941*. (a, b) IncuCyte^®^ (Sartorius, Göttingen, Germany) proliferation assay of (a) Met-1 and (b) IC1 Met cutaneous squamous cell carcinoma. Each cell line was transfected with three different *LINC00941* targeting antisense oligonucleotide gapmers (G1–G3) and one scrambled noncoding control (negative control). Each transfection was repeated 3 times across both cell lines and 6 technical growth replicates of 1000 cells were seeded and imaged over 7 days, scanning every 24 h. Statistics correspond to Anova of confluency values relative for each gapmer treatment to negative control. (c, d) Quantitative reverse transcription polymerase chain reaction relative fold expression of *LINC00941* in (c) Met-1 and (d) IC1 Met cells. Student’s *t*-tests were performed relative for each gapmer treatment to negative control; three biologic transfection replicates of each cell line were the same as used in the proliferation assays. (e–g) Relative colony numbers of (f) Met-1 and (g) IC1 Met cell lines taken over a 14-day period. *P*-values were calculated using a Student’s *t*-test; three biologic transfection replicates of each cell line were the same used in proliferation assays. Representative colony-formation images of IC1 Met and Met-1 cells from one biologic replicate (e). ns, not statistically significant (i.e. *P* > 0.05). *P < 0.05, **P < 0.01, ***P < 0.001, ****P < 0.0001.

The lncRNAs in the brown eigengene module were associated with several pathways linked to proliferation (Figure 3c). As many of these are expressed at a high level in cSCC cell lines (Figure 4b), we reasoned that they are likely to play functionally important roles in cellular proliferation. We investigated the expression level of *LINC00941* as a potential pro-proliferative lncRNA and found that its expression levels increased with cSCC disease state and that it was expressed at a high level in our cSCC PDCL panel (Figure 5a). Furthermore, high *LINC00941* expression levels correlated with poor patient survival and a high sample progenitor score in the HNSC and PAAD datasets [Figure 5b; Figure S10 (see Supporting Information)]. Furthermore, we found that *LINC00941* was co-expressed with a variety of oncogenes, including – most significantly – *HMGA2* (Table S9, Figure S11; see Supporting Information).^52^ We inhibited *LINC00941* expression in the Met-1 and IC1 Met PDCLs using three different gapmer antisense oligonucleotides and measured the effects of doing so on cell proliferation in IncuCyte (Sartorius) proliferation and colony-forming assays *in vitro*. We observed a significant reduction in cell proliferation over 7 days, with gapmer1 (G1) having the most profound reduction in growth in both cell lines (Figure 6a, b). Colony-forming capacity was also inhibited to a similar extent following gapmer treatment (Figure 6e–g). qRT-PCR revealed that the inhibition of proliferation and colony formation was largely dose-dependent, with G1 treatment exhibiting the largest effect on proliferation and colony formation, and the strongest inhibition of *LINC00941* expression. Gapmer3 treatment had the least inhibitory effect on *LINC00941* expression and the smallest effect on cell proliferation and colony-forming potential (Figure 6c, d).

## Discussion

Despite being one of the most prevalent malignancies worldwide, late-stage cSCC has limited targeted treatment options and remains a significant burden, particularly for people who are immunocompromised.^53^ Much remains to be learned about the molecular basis of cSCC disease progression and the functional roles of the vast majority of the estimated 100 000 lncRNAs.^34,54^ Initial studies have highlighted the potential of lncRNAs as functionally relevant regulators of cancer, with *MALAT1* providing the paradigm example.^55^ Subsequently, lncRNAs have been associated with hallmarks of cancer and identified as being differentially expressed in all cancer types, including cSCC.^56–58^ The potential relevance of lncRNAs as clinical targets in cSCC has not gone unnoticed. Bulk RNAseq was used to explore noncoding transcriptomic changes in two previous studies,^44,59^ revealing multiple potential modulators of disease progression. Both of these important studies have reinforced the idea that lncRNAs have unmet therapeutic potential and warrant further investigation in cSCC. However, the studies were potentially limited by their small sample sizes. Here, we analysed mRNA expression – including lncRNA expression – in 110 patient samples profiled by RNAseq,^3^ incorporating RNAseq data from 15 PDCLs and investigating lncRNA correlates in 10 previous RNAseq datasets, to generate the most comprehensive resource for predicting lncRNA functions in cSCC to date.

Our analysis revealed correlations of lncRNA and mRNA expression with disease progression, enabling us to generate a 267 lncRNA gene expression signature that correlated with differentiation to a progenitor-like status in cSCC and poor patient outcomes in HNSC and PAAD. We selected *LINC00941* for proof-of-principle *in vitro* validation of our eigengene functional correlations and revealed that this lncRNA promotes cancer cell proliferation, as predicted by the correlation analysis. As we have shown that the biomarker potential of this lncRNA gene set is not limited to cSCC through our analysis in HNSC and PAAD, the roles predicted for the functionally relevant molecules identified may go beyond cSCC and provide a background for future studies in similar disease types.

The specific lncRNA highlighted in this study (i.e. *LINC00941*) was selected primarily for its association with pro-proliferative pathways and its abundance in IC1 Met and Met-1 cell lines; it has also been found to have roles in various tissue types and cancers. In pancreatic cancer, *LINC00941* has been associated with increased cellular proliferation and invasiveness via sponging the microRNA miR-335-5p,^60^ and in glioma the upregulation of *LINC00941* has been identified as having the same effects through unknown mechanisms.^61^ In skin, *LINC00941* has also been identified as a regulator of normal keratinocyte differentiation through interacting with metastasis-associated protein 2 (MTA2/NuRD),^62^ the deregulation of which is also linked to cSCC development. We found that *LINC00941* was not only positively correlated with proliferative pathways in cSCC, but also that it directly contributes to the *in vitro* proliferation and colony-formation capabilities of PDCLs. This highlights both the importance of *LINC00941* as a potential therapeutic target, and the power of the suite of correlation analyses performed in this study for identifying candidate lncRNA genes.

With limited late-stage targeted therapeutic options available for cSCC, we believe the identification of largely uncharacterized lncRNA associated with various regulatory roles in disease progression described here warrant further investigation.

## Supplementary Material

ljaf108_Supplementary_Data

## Data Availability

The patient-derived cutaneous squamous cell carcinoma cell line RNA sequencing data have been deposited in the Gene Expression Omnibus (accession code GSE266912; https://www.ncbi.nlm.nih.gov/geo/query/acc.cgi?acc=GSE266912). All other data are provided in the Supporting Information and code is available on request.
