# Supplementary material for: The landscape of long noncoding RNA during cutaneous squamous cell carcinoma progression
Source: Br J Dermatol. 2025 Mar 27;193(3):490–501. doi: 10.1093/bjd/ljaf108 (PMC12360040; doi:10.1093/bjd/ljaf108)
Supplement: ljaf108_Supplementary_Data [file ljaf108_supplementary_data.zip › Supplementary file.docx]

***Appendix S1***

**Supplementary** **materials and methods**

**RNA Sequencing**

Samples derived from Bailey et.al.^1^ included; normal skin (n=26), AK (n=14), primary tumour (n=66), and metastatic cSCC (n=4). In summary, sequencing libraries were prepared using the TruSeq Stranded Total RNA protocol (ribo-zero) (part no. 15031048 Rev. D April 2013) (illumina), starting with 500ng-1ug total RNA. In brief, the RNAseq libraries underwent sequencing on the HISeq2000 (illumina) platform. Subsequently, the fastq files were aligned using the RNAseq nf-core pipeline. Raw read data were trimmed for adapters using trimagalore and aligned with the GRCh38 human reference genome assembly using STAR aligner^2,3^. Samples were analysed for quality using FASTQC and those not meeting the cut-offs were excluded. Normalisation, differential gene expression and statistical analysis were carried out using the R packages edgeR, limma, and DESeq2 using standard workflows^4,5,6^. Long non coding RNAs were categorised from alternative RNA subgroups using the R package biomaRt^7^. The packages ggplot2 and ComplexHeatmaps were used for all plotting and statistics were generated using ggpubr^8,9^. Full informatic analysis workflow is available in supplementary methods.

***Correlative analysis***

Tanglegrams were generated using the R package dendextend^10^, Uniform Manifold Approximation and Projection (UMAP) plots were generated using the R package umap derived from the python software UMAP^11^. ConsensusClusterPlus was used for the generation of k-means clustered groups that could subsequently be used for the generation of a lncRNA signature score^12^. Gene groupings across multiple sample conditions visualised using upset plots were generated in R using upsetR^13^. Weighted correlation network analysis (WGCNA) was performed for clustering as per the R package protocol with soft thresholding powers of 14 being selected^14^.

***Survival analysis***

Gene expression and survival data were obtained from The Cancer Genome Atlas (TCGA Pan-Cancer [https://www.cancer.gov/tcga](https://www.cancer.gov/ccg/research/genome-sequencing/tcga)), realigned to incorporate the most recent lncRNA transcripts by the recount3 project^15^. Plotting was carried out using the survplot function of the package rms and the survival package in RStudio^16,17^. *LINC00941* survival and statistical analysis was carried out on the online platform GEPIA^18^.

***Tissue Culture***

Human IC1 Met (Cancertools.org Cat. #153676) and MET-1 (RRID:CVCL LN09) SCC cell lines were previously authenticated and established to passage 12 and tested for mycoplasma contamination with the Venor GeM qOneStep Mycoplasma detection kit (Minerva Biolabs). Further details of these cell lines are found in Hassan et.al. 2019^19^. Cells were cultured in a keratinocyte medium (a 3:1 V/V mixture of DMEM and Ham’s F12 (Gibco, ThermoFischer) supplemented with 10% FBS and a cocktail of mitogens: 0.4 µg/mL hydrocortisone, 10–10 M choleratoxin, 5 µg/mL transferrin, 2 × 10–11 M liothyronine, 5 µg/mL insulin, 10 ng/mL epidermal growth factor (EGF), as described in depth in Hassan et.al. 2019^19^.

***Antisense oligonucleotide knockdown***

Customised antisense oligonucleotides (GAPMERs) targeting *LINC00941*, GAPMER ID 1 (LG00796994), GAPMER ID 2 (LG00796996), GAPMER ID 3 (LG00796997)^20^ were designed by Qiagen. Cells were cultured as previously described in 6 well plates to a confluency of 70-80%, aspirated, washed twice with PBS and transfected with 25nM GAPMER and 25nM lipofectamine 2000 (Thermofisher) in optiMEM medium (Thermofisher) for 48hrs. Transfection medium was then aspirated, cells were washed twice with PBS and previously described cell culturing keratinocyte medium was added.

***qRT-PCR***

qRT-PCR was performed using SYBR green (ThermoFisher) on a Quantstudio3 machine (ThermoFisher) on the ΔΔCT method as per the manufacturer’s protocol. Primers for *LINC00941* forward (GACCTTTTCAGGCCAGCATT) and reverse (ACAATCTGGATAGAGGGCTCA) and *GAPDH* forward (GGATTTGGTCGTATTGGG) and reverse (GGAAGATGGTGATGGGATT), were purchased from ThermoFisher. GraphPad Prism v10 (GraphPad, Inc., USA), was used to generate figures and conduct Mann-Whitney U-tests.

***Growth assays***

For proliferation assays, 24hrs post transfection cells were split at a density of 1000 cells in 6 replicates in a 96 well flat bottomed plate (Corning) and imaged using an IncuCyte® ZOOM Live-Cell Analysis System for 7 days. All proliferation assays were carried out in the same conditions as cell culturing with 5% CO2 at 37°C. For colony formation assays 100 cells were cultured in a 6 well flat bottomed plate (Corning) for 14 days, fixed with 2% crystal violet in methanol, then counted under a Zeiss Axiostar light microscope.

All statistical analysis was conducted in GraphPad Prism v10 (GraphPad, Inc., USA), with a Mann-Whitney U-test performed on colony formation data and end points of proliferation data relative to -ve controls.

***Supplementary figures***


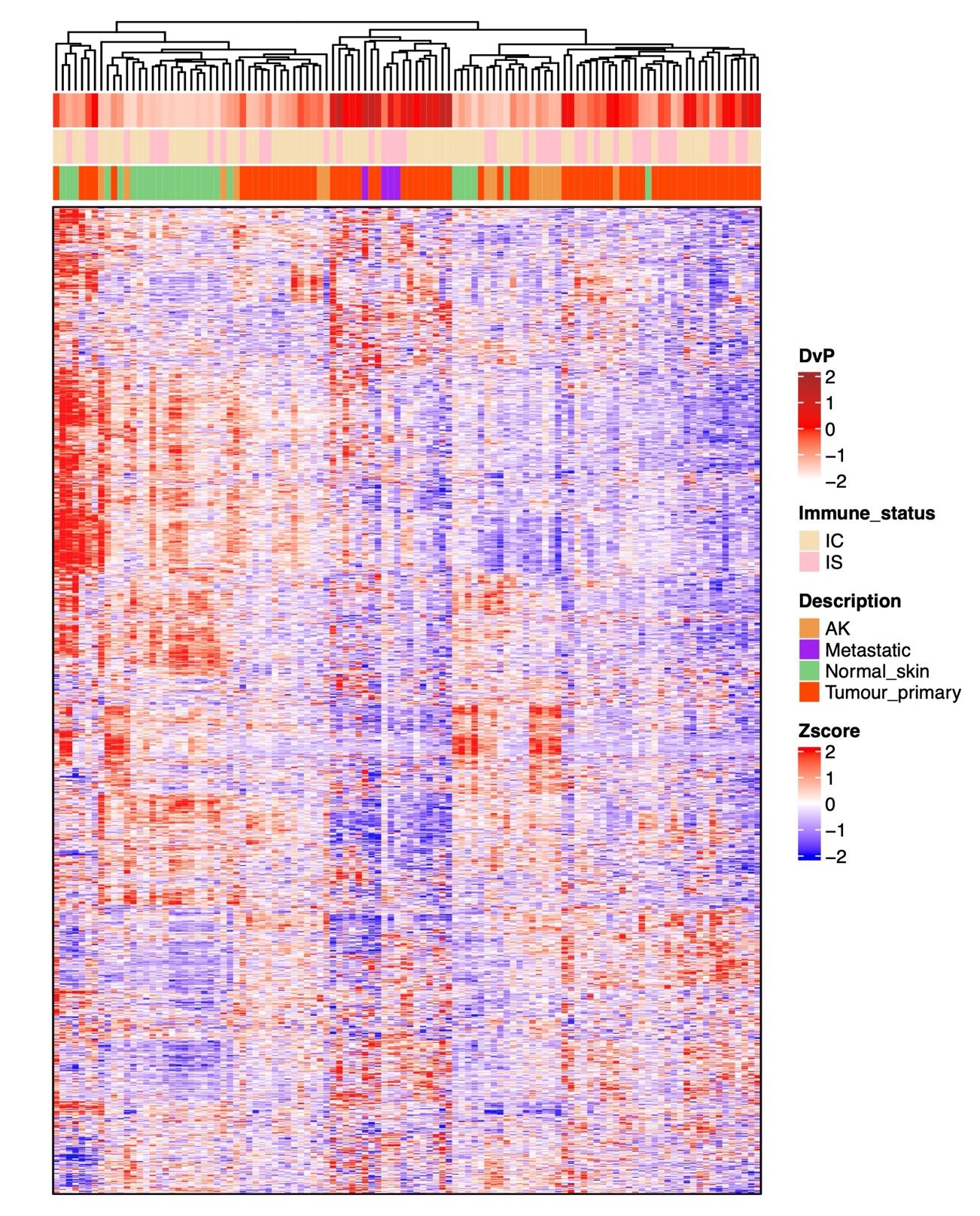
 **Supplementary figure 1.** **Unsupervised clustering of lncRNA expression in cSCC.** A) heatmap of lncRNA expression in cSCC was generated in R using ComplexHeatmaps, clustering in an unsupervised manner between samples. A differentiated versus progenitor score (DvP) indicates with a higher score a higher degree of progenitor like state developed from Bailey et.al 20231. The immune status of each sample was also included with IC = immune competent and IS = immune suppressed, normal skin (n=26), actinic keratoses (n=14), primary tumour (n=66), and metastatic cSCC (n=4).


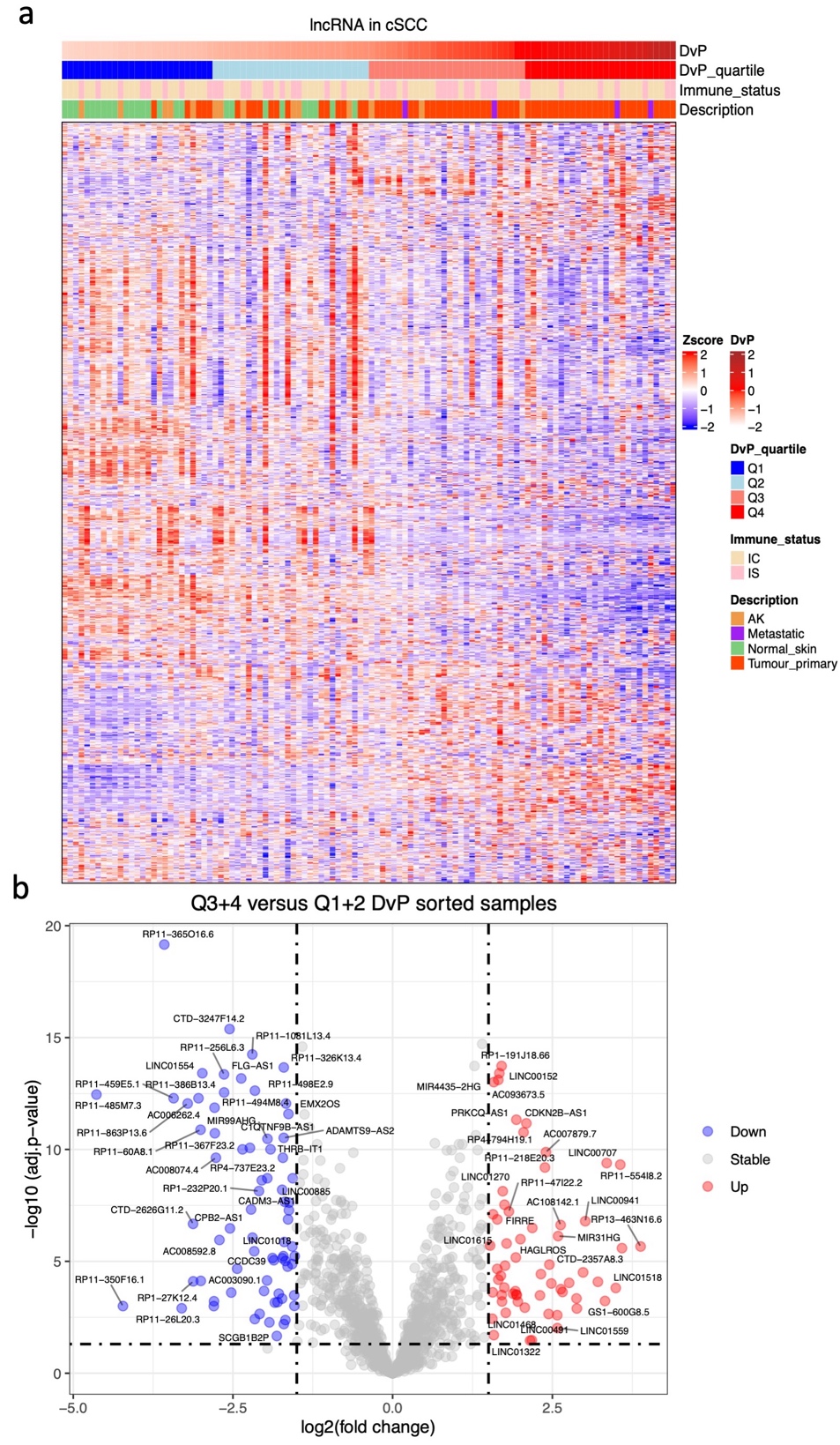


**Supplementary figure 2. Progenitor score ranked lncRNA expression in cSCC** (A) A heatmap of lncRNA expression in cSCC was generated in R using ComplexHeatmaps and ranked based on the previously generated DvP progenitor score^1^. A differentiated versus progenitor score (DvP) indicates with a higher score a higher degree of progenitor like state, quartiles were ranked on DvP score (Q1 n = 27, Q2 n = 28, Q3 n = 27, Q4 n = 28). The immune status of each sample was also included with IC = immune competent and IS = immune suppressed. B) Volcano plots of log2 fold change (log2FC) against log10 of the adjusted P value of the degree in change showing lncRNA expression alterations only. Up (red dots) denotes an upregulation of gene expression derived from a log2FC > 1.5 and a significance of p< 0.05. Down (blue dots) denotes downregulated gene expression log2FC < -1.5 and significance of p< 0.05. Stable (grey dots) was defined as all genes whose expression did not meet “up” or “down” criteria. Samples comparisons were made between a combined Q3+4 vs Q2+1 sample cohort to measure high vs low ranked DvP samples.


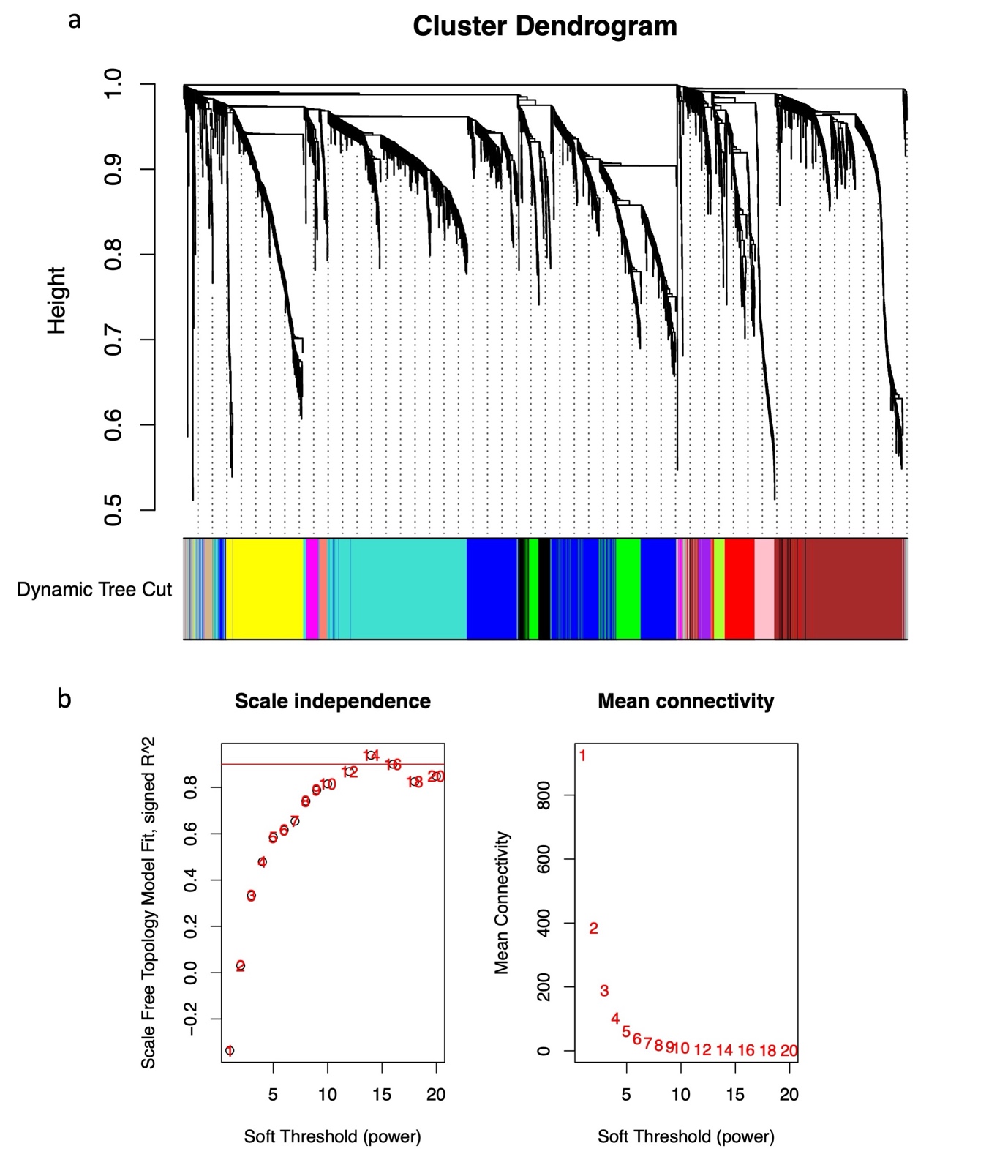


**Supplementary figure 3.** **WGCNA parameters.** A) Hierarchical clustering dendrogram of all genes and correspondingly coloured eigengene module generation, generated through the WGCNA package in R using a power of 14 and a Spearman’s correlation to generate a topological overlap matrix. B) The soft thresholding parameters set for beginning a WGCNA, a power of 14 was selected based on the scale independence and mean connectivity of the data.


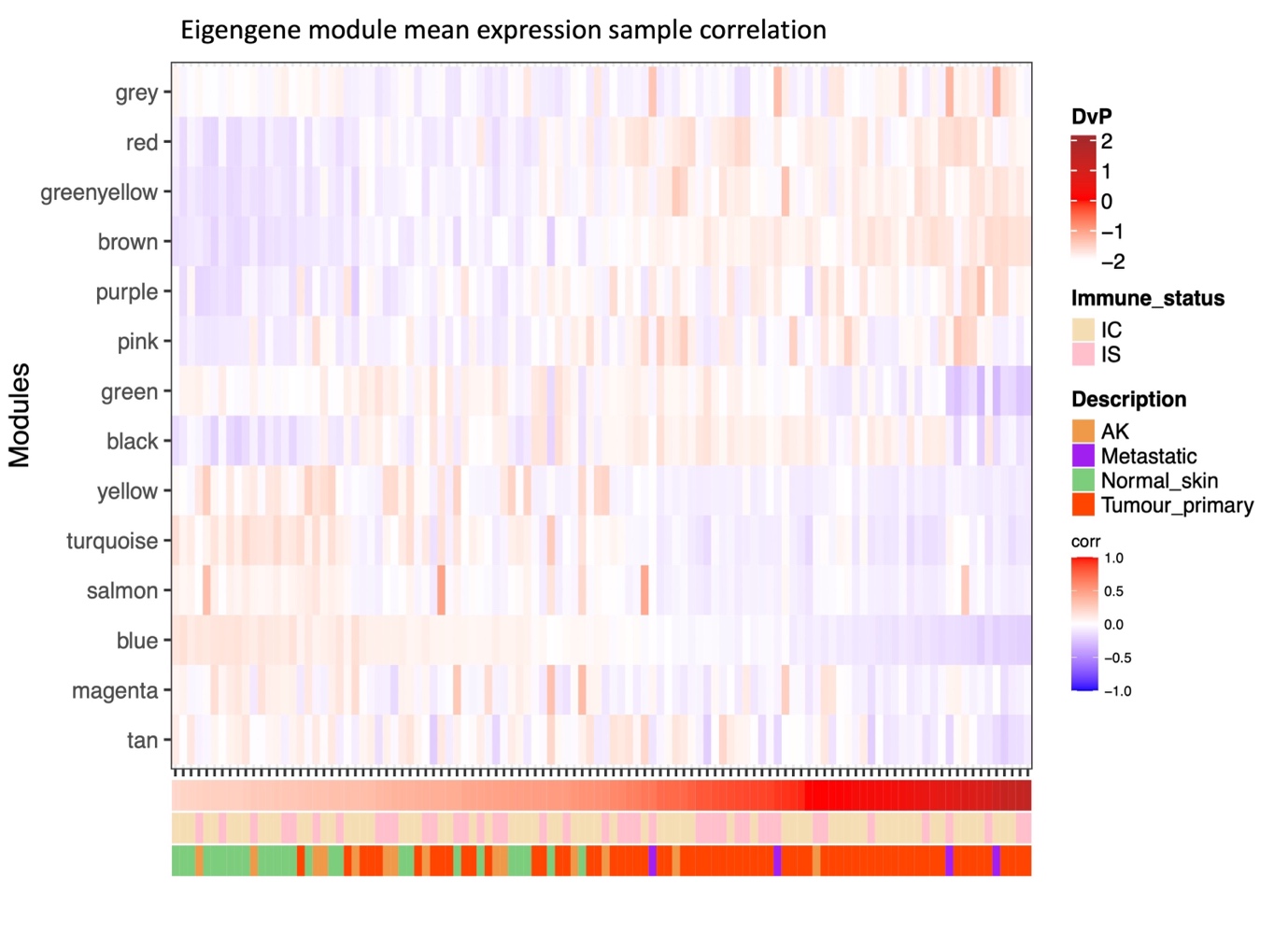


**Supplementary figure 4. Weighted correlation network analysis (WGCNA) of differentially expressed genes.** The entire gene list of genes present in Fig 2G was used for the WGCNA analysis. Collapsed eigengene module expression correlated to DvP score, corr = Spearman’s correlation of each eigengene module to each sample. The immune status of each sample was also included with IC = immune competent and IS = immune suppressed. A differentiated versus progenitor score (DvP) indicates with a higher score a higher degree of progenitor like state. Colour named groups refer to the eigengene cluster defined by Supplementary Figure 2 parameters.


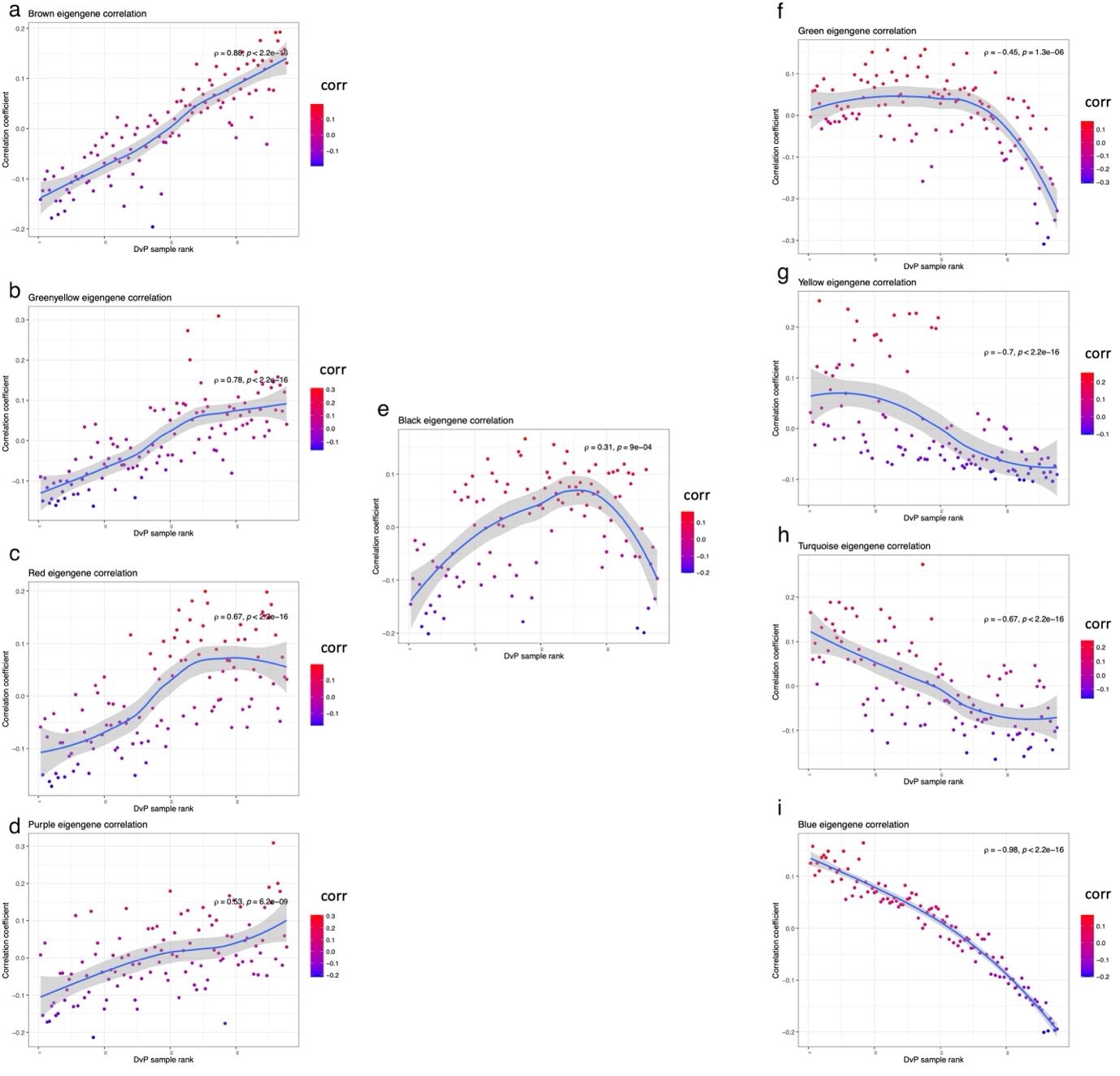


**Supplementary figure 5. The correlation of eigengene modules to DvP score.** A-I) The significantly correlated eigengene modules generated from WGCNA. Spearman’s correlation coefficient of each eigengene module to cSCC samples ranked on the DvP axis. Spearman’s rho (ρ), and P values (limited to 2.2e-16) are displayed on the plot as is a generalised additive model line fitted using R. Value colouring of each point corresponds to correlation coefficients.


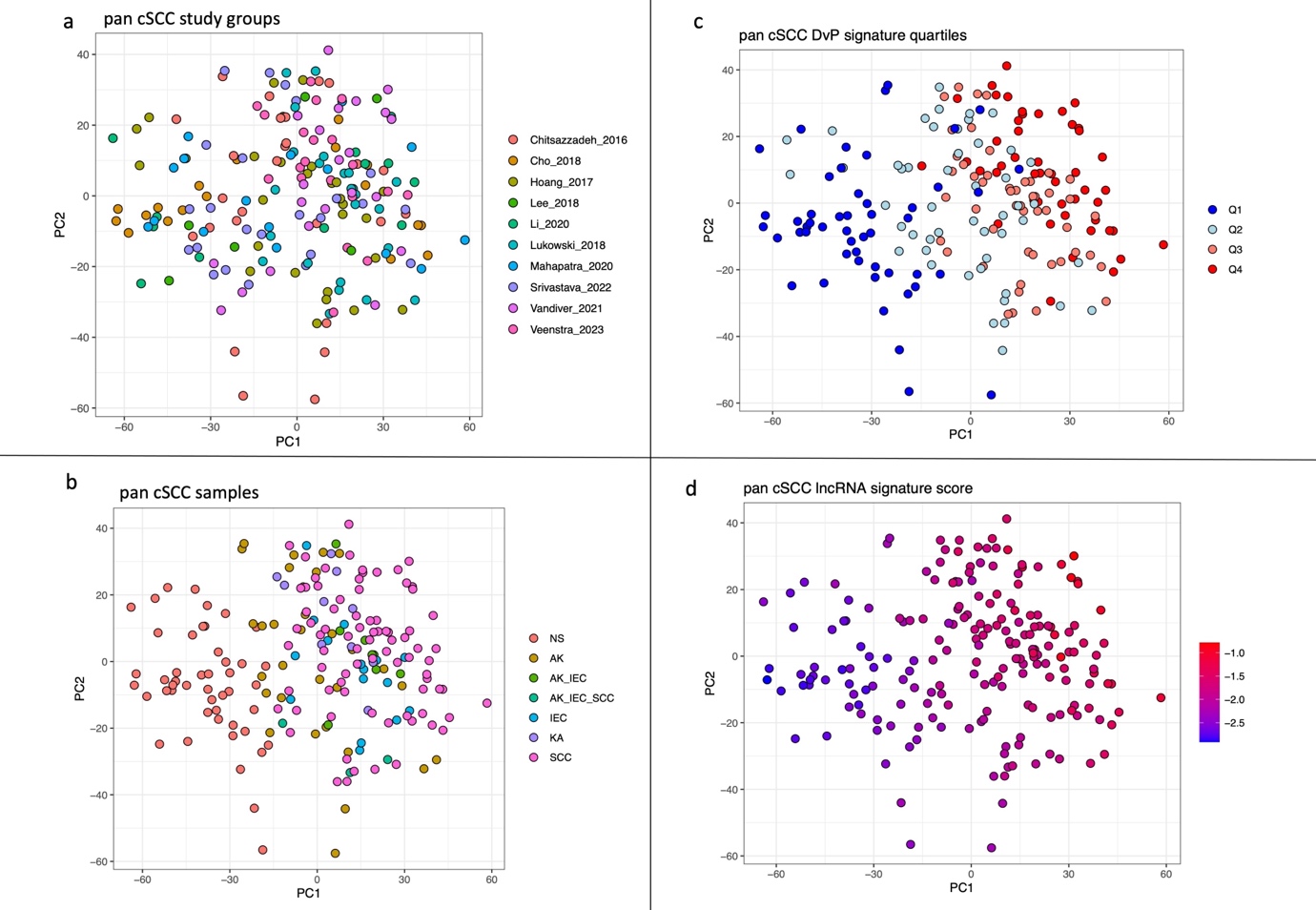


**Supplementary figure 6. Analysis of lncRNA signature genes in alternative cSCC cohorts.** 10 alternative cSCC cohorts that had previously been batch normalised were obtained (detailed in Methods section) and subjected to multiple PCA analyses. A) Denotes the unsupervised clustering of samples based on total gene expression coloured by the specific RNAseq cohort. B) Denotes the unsupervised clustering of samples based on total gene expression coloured by the specific clinically defined sample condition (NS = normal skin, AK = actinic keratoses, AK_IEC = actinic keratoses/in situ intraepidermal carcinomas, AK_IEC_SCC = actinic keratoses/in situ intraepidermal carcinomas/squamous cell carcinoma, IEC = in situ intraepidermal carcinomas, KA = keratoacanthomas, SCC = squamous cell carcinoma). C) Denotes the unsupervised clustering of samples based on total gene expression coloured by the DvP signature scored quartile of each sample Q1 = lowest DvP score Q4 = highest. D) Denotes the unsupervised clustering of samples based on total gene expression coloured by lncRNA signature derived from the 267 genes.


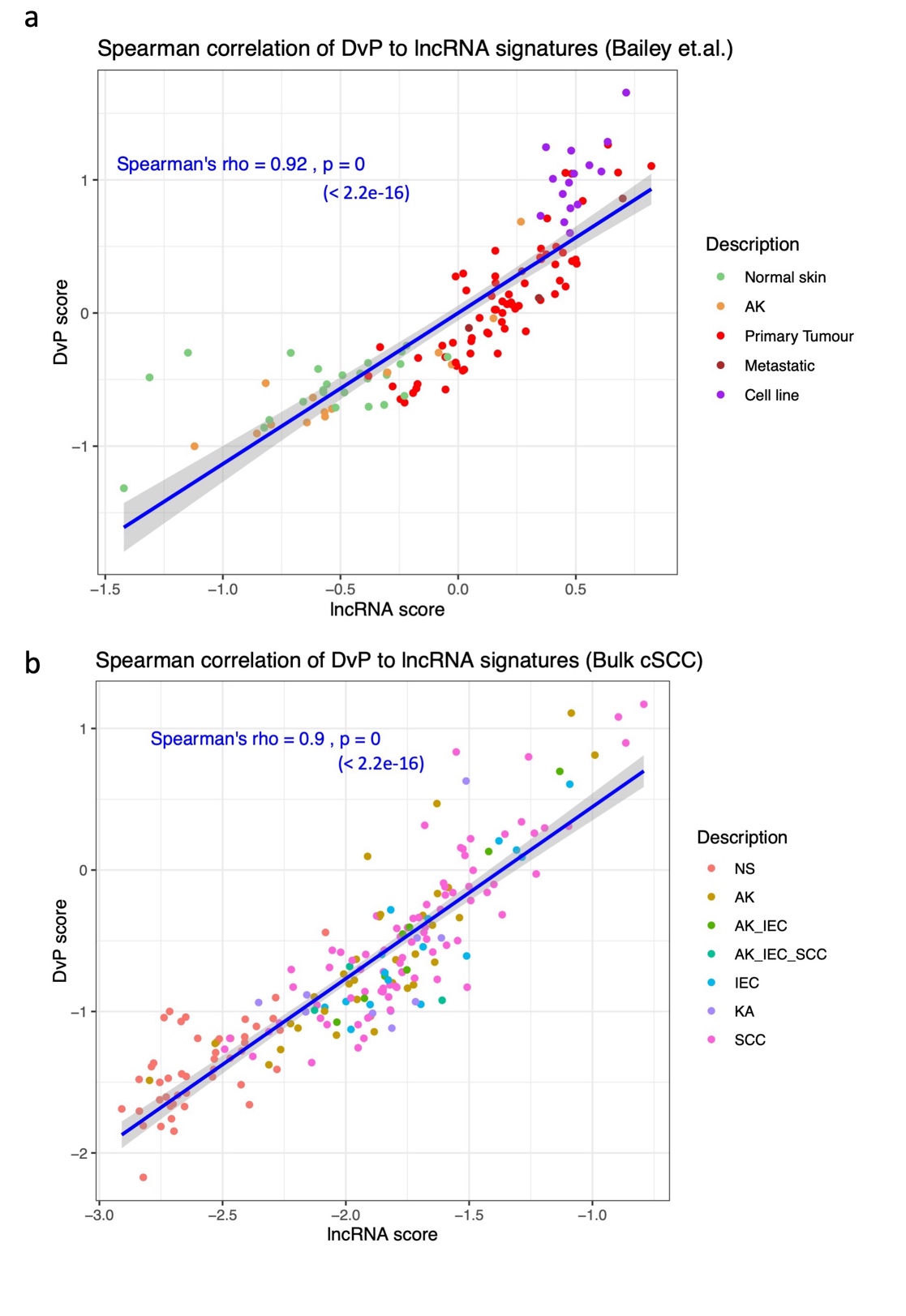


**Supplementary figure 7. The relationship of DvP and lncRNA signature scores across cSCC cohorts** A) The Spearman’s correlation coefficient and p value for each of the 110 patient-derived samples and 15 PDCLs’ relative lncRNA signature score relative to the DvP signature score. A p value of 0 is denoted as it was calculated to be < 2.2e-16. A) The Spearman’s correlation coefficient and p value of each sample in the 10 alternative cSCC cohorts that had previously been batch normalised (detailed in Methods section) relative lncRNA signature score relative to the DvP signature score. A p value of 0 is denoted as it was calculated to be < 2.2e-16 (NS = normal skin, AK = actinic keratoses, AK_IEC = actinic keratoses/in situ intraepidermal carcinomas, AK_IEC_SCC = actinic keratoses/in situ intraepidermal carcinomas/squamous cell carcinoma, IEC = in situ intraepidermal carcinomas, KA = keratoacanthomas, SCC = squamous cell carcinoma).


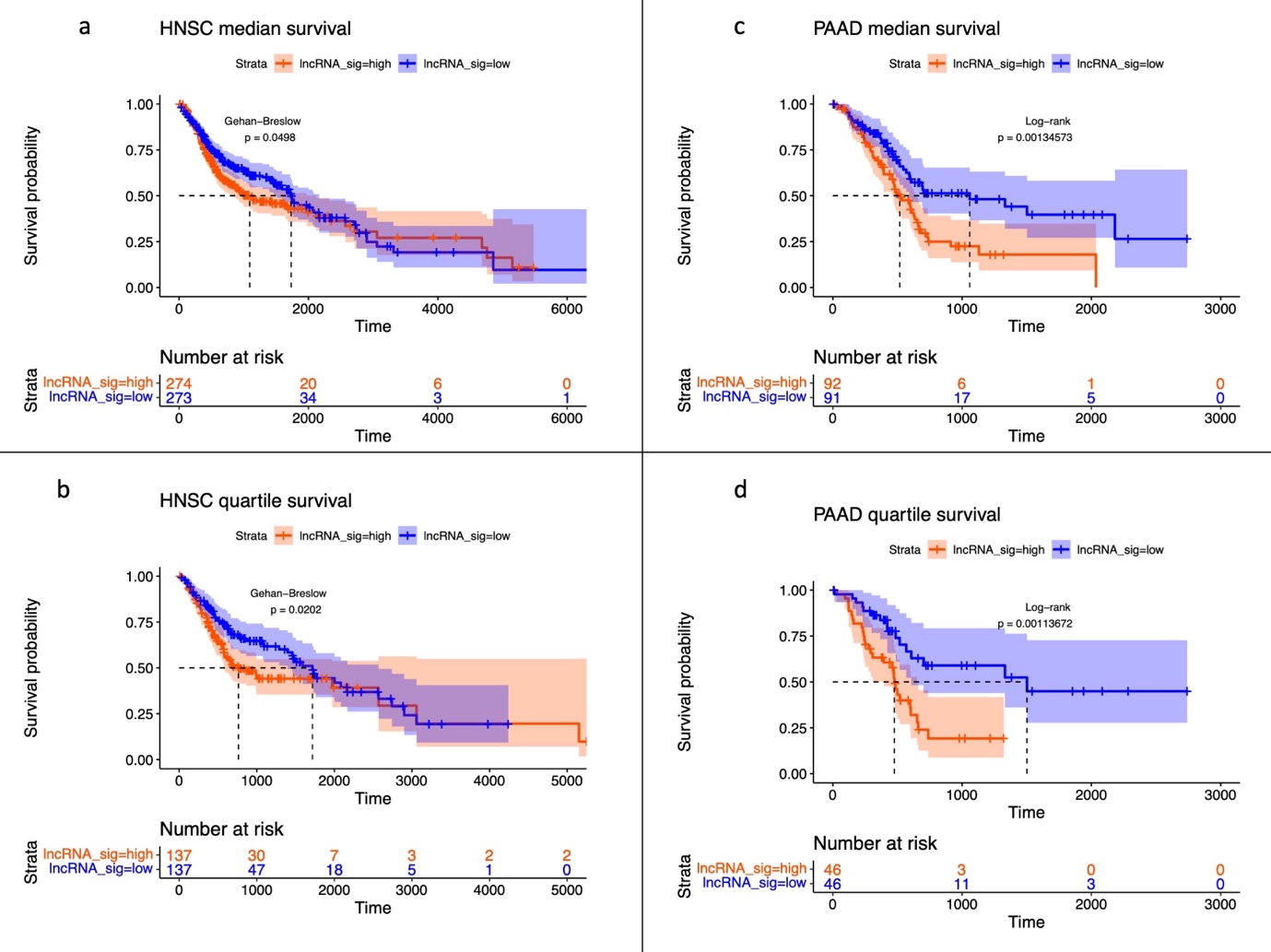


**Supplementary figure 8. Predicting the survival of patients with HNSC and PAAD using a generated lncRNA signature.** A lncRNA signature score was used in conjunction with TCGA datasets reanalysed by the recount3 project to predict patient outcome in alternative cancer types^1^. The survival probability was calculated as the number of subjects surviving divided by the number of patients at risk^2^ A) The median survival data for HNSC. B) The quartile survival data for HNSC. C) The median survival data for PAAD. D) The median survival data for PAAD.


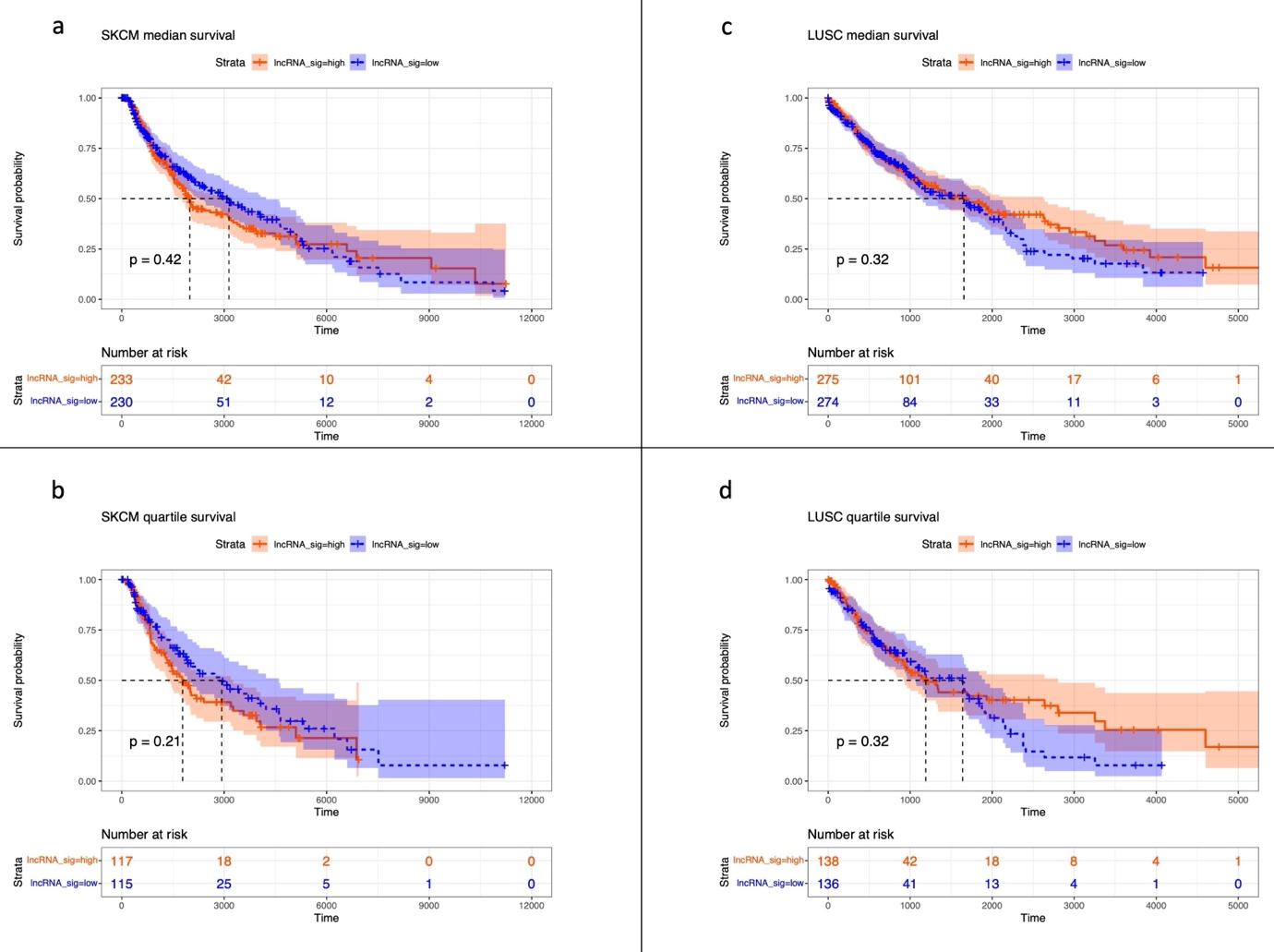


**Supplementary figure 9. Predicting the survival of patients with SKCM and LUSC using the lncRNA signature**. A lncRNA signature score was used in conjunction with TCGA datasets reanalysed by the recount3 project to predict patient outcome in alternative cancer types^1^. The log-rank p value was calculated for each and the survival probability was calculated as the number of subjects surviving divided by the number of patients at risk^2^ A) The median survival data for SKCM. B) The quartile survival data for SKCM. C) The median survival data for LUSC. D) The median survival data for LUSC.


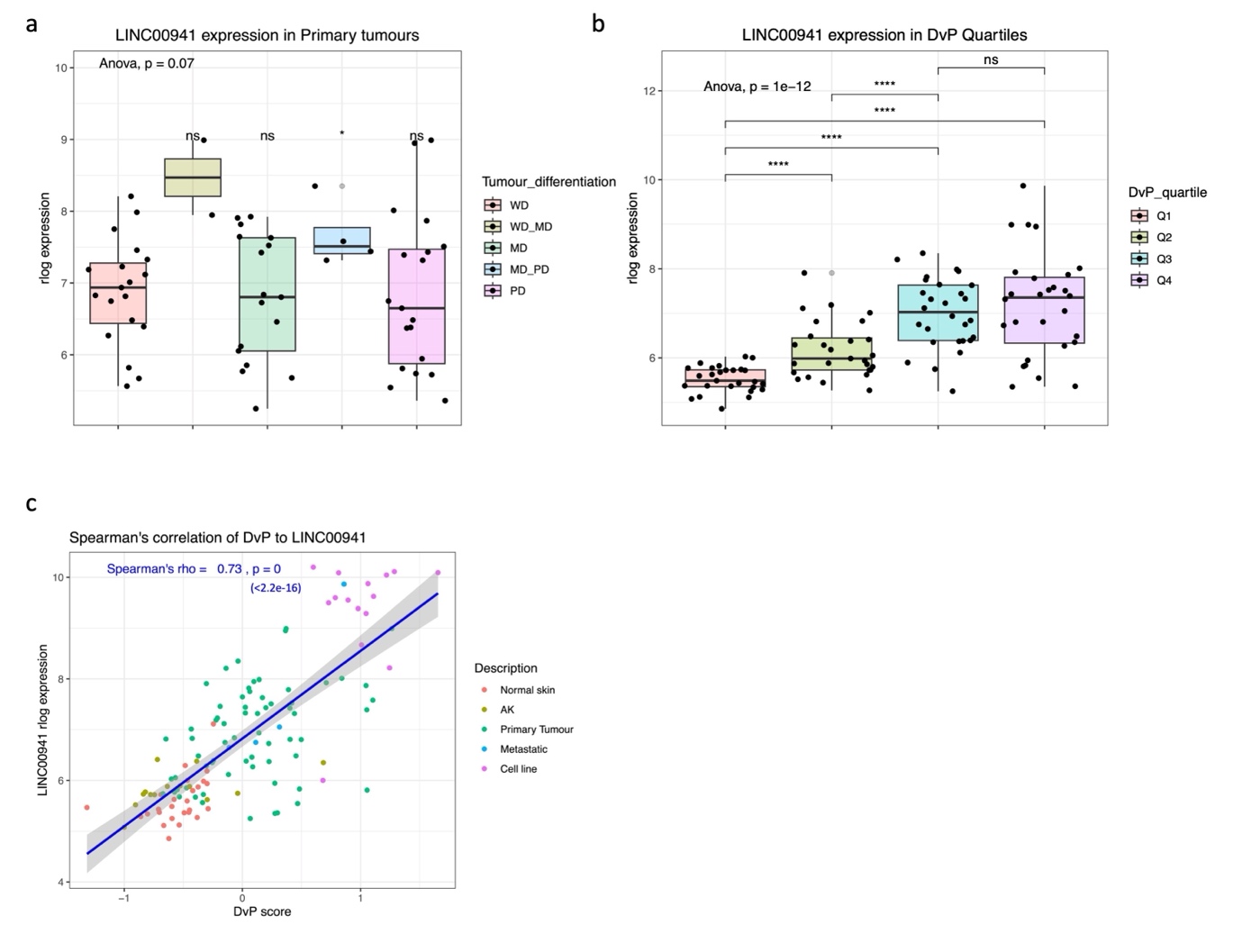


**Supplementary figure 10. The relation of LINC00941 to differentiation status in cSCC.** A) Boxplot of the rlog expression of LINC00941 in primary tumour samples only, grouped by clinically determined differentiation status (WD = well differentiated, WD_MD = well differentiated/moderately differentiated, MD = moderately differentiated, MD_PD = moderately differentiated/poorly differentiated, PD = poorly differentiated). Statistical comparisons were derived from a student’s t-test against WD samples. B) Boxplot of LINC00941 rlog expression in 110 DvP quartile ranked patient samples (Q1 = most differentiated (lowest score), Q4 = most progenitor (highest score)). C) Spearman’s correlation of LINC00941 rlog expression and relative DvP score of all samples including 15 PDCLs. (ns = P > 0.05, * = P < 0.05, ** = P < 0.01, *** = P < 0.001, **** = P < 0.0001).


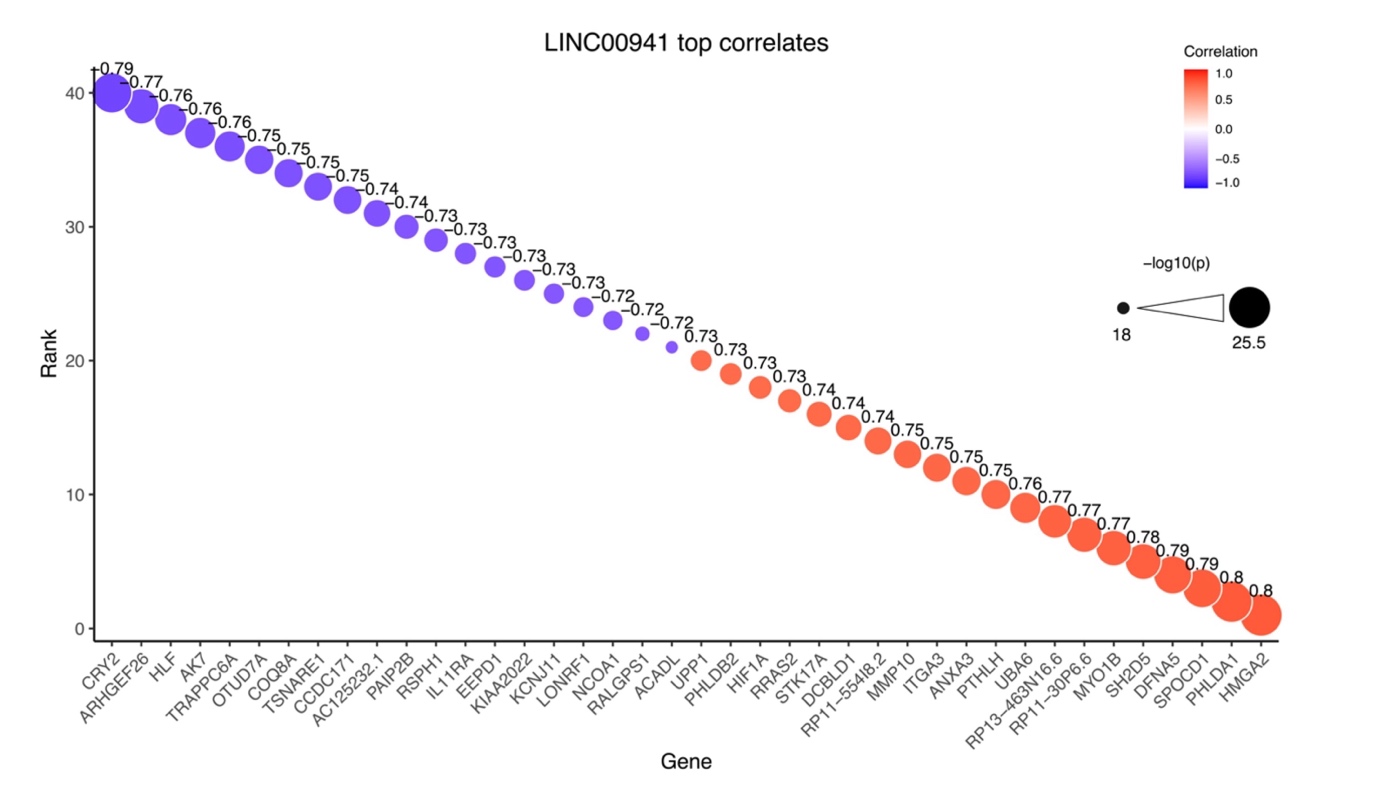


**Supplementary figure 11. Visualisation of top LINC00941 coexpressed genes.** The top and bottom 20 most significantly Spearman’s correlated genes to LINC00941 rlog expression were obtained from combined Bailey et.al.^1^ and 15 PDCL RNAseq datasets. Size corresponds to log10(p. Val), colour to Spearman’s cor value, gene ranks were given inversely to correlation coefficients.

**References**

1. Bailey, P. *et al.* Driver gene combinations dictate cutaneous squamous cell carcinoma disease continuum progression. *Nat. Commun.* **14**, 5211 (2023).

2. Ewels, P. A., Peltzer, A., Fillinger, S., Patel, H., Alneberg, J., Wilm, A., Garcia, M. U., Di Tommaso, P. & Nahnsen, S. The nf-core framework for community-curated bioinformatics pipelines. *Nat Biotechnol* 38, 276–278 (2020).

3. Dobin, A., Davis, C. A., Schlesinger, F., Drenkow, J., Zaleski, C., Jha, S., Batut, P., Chaisson, M. & Gingeras, T. R. STAR: ultrafast universal RNA-seq aligner. *Bioinformatics* 29, 15–21 (2013).

4. Love, M. I., Huber, W. & Anders, S. Moderated estimation of fold change and dispersion for RNA-seq data with DESeq2. *Genome Biol* 15, 1–21 (2014).

5. Ritchie, M. E., Phipson, B., Wu, D., Hu, Y., Law, C. W., Shi, W. & Smyth, G. K. Limma powers differential expression analyses for RNA-sequencing and microarray studies. *Nucleic Acids Res* 43, e47 (2015).

6. Robinson, M. D., McCarthy, D. J. & Smyth, G. K. edgeR: A Bioconductor package for differential expression analysis of digital gene expression data. *Bioinformatics* 26, 139–140 (2009).

7. Smedley, D., Haider, S., Ballester, B., Holland, R., London, D., Thorisson, G. & Kasprzyk, A. BioMart - Biological queries made easy. *BMC Genomics* 10, 1–12 (2009).

8. Gu, Z., Eils, R. & Schlesner, M. Complex heatmaps reveal patterns and correlations in multidimensional genomic data. *Bioinformatics* 32, 2847–2849 (2016).

9. Wickham, H. Ggplot2. *Wiley Interdiscip Rev Comput Stat* 3, 180–185 (2011).

10. Galili, T. dendextend: an R package for visualizing, adjusting and comparing trees of hierarchical clustering. *Bioinformatics* 31, 3718–3720 (2015).

11. McInnes, L., Healy, J. & Melville, J. UMAP: Uniform Manifold Approximation and Projection for Dimension Reduction. (2018).

12. Wilkerson, M. D. & Hayes, D. N. ConsensusClusterPlus: A class discovery tool with confidence assessments and item tracking. *Bioinformatics* 26, 1572–1573 (2010).

13. Conway, J. R., Lex, A. & Gehlenborg, N. UpSetR: An R package for the visualization of intersecting sets and their properties. *Bioinformatics* 33, 2938–2940 (2017).

14. Langfelder, P. & Horvath, S. WGCNA: An R package for weighted correlation network analysis. *BMC Bioinformatics* 9, (2008).

15. Wilks, C., Zheng, S. C., Chen, F. Y., Charles, R., Solomon, B., Ling, J. P., Imada, E. L., Zhang, D., Joseph, L., Leek, J. T., Jaffe, A. E., Nellore, A., Collado-Torres, L., Hansen, K. D. & Langmead, B. recount3: summaries and queries for large-scale RNA-seq expression and splicing. *Genome Biol* 22, 1–40 (2021).

16. Harrell, F. E. Regression Modeling Strategies. (2023).

17. Lumley, T., S-, R., Elizabeth, A., Cynthia, C. & Therneau, M. T. M. Package ‘ survival ’. (2024).

18. Tang, Z., Li, C., Kang, B., Gao, G., Li, C. & Zhang, Z. GEPIA: a web server for cancer and normal gene expression profiling and interactive analyses. *Nucleic Acids Res* 45, W98–W102 (2017).

19. Hassan, S., Purdie, K. J., Wang, J., Harwood, C. A., Proby, C. M., Pourreyron, C., Mladkova, N., Nagano, A., Dhayade, S., Athineos, D., Caley, M., Mannella, V., Blyth, K., Inman, G. J. & Leigh, I. M. A Unique Panel of Patient-Derived Cutaneous Squamous Cell Carcinoma Cell Lines Provides a Preclinical Pathway for Therapeutic Testing. *Int J Mol Sci* 20, (2019).

20. Lee, J.-S. & Mendell, J. T. Antisense-Mediated Transcript Knockdown Triggers Premature Transcription Termination. *Mol Cell* 77, 1044-1054.e3 (2020).
